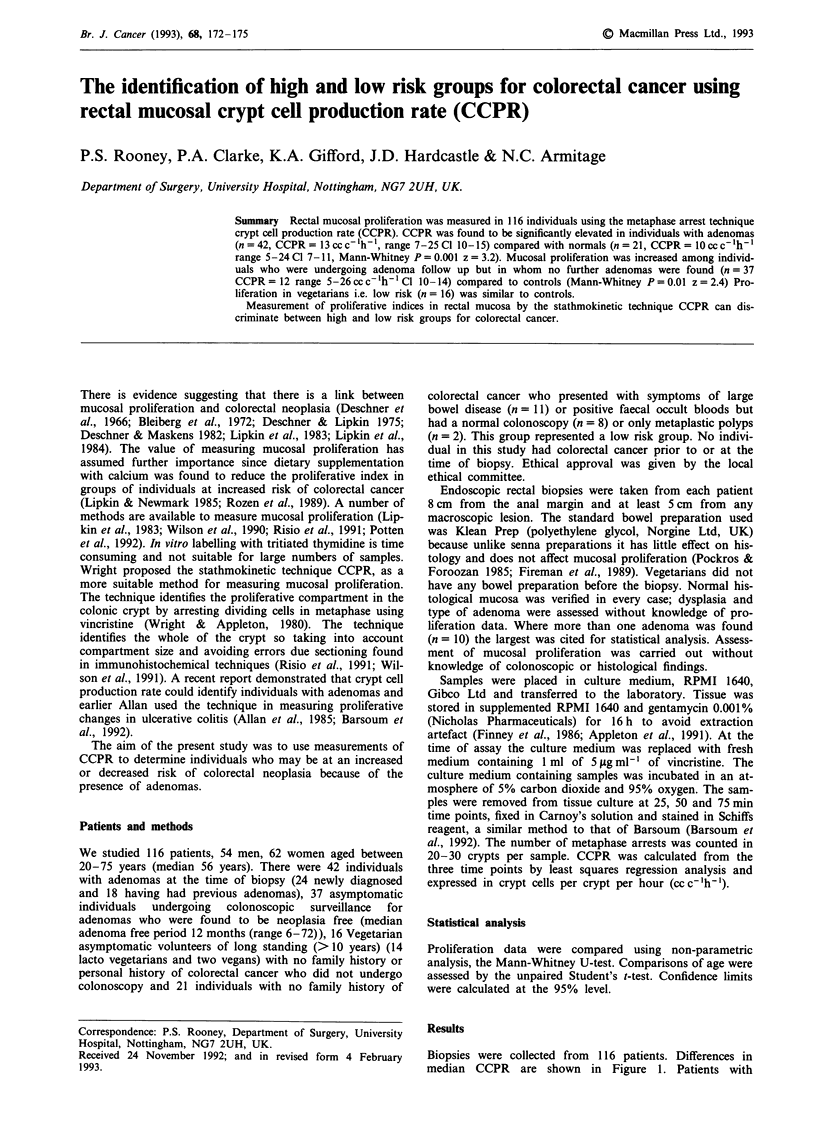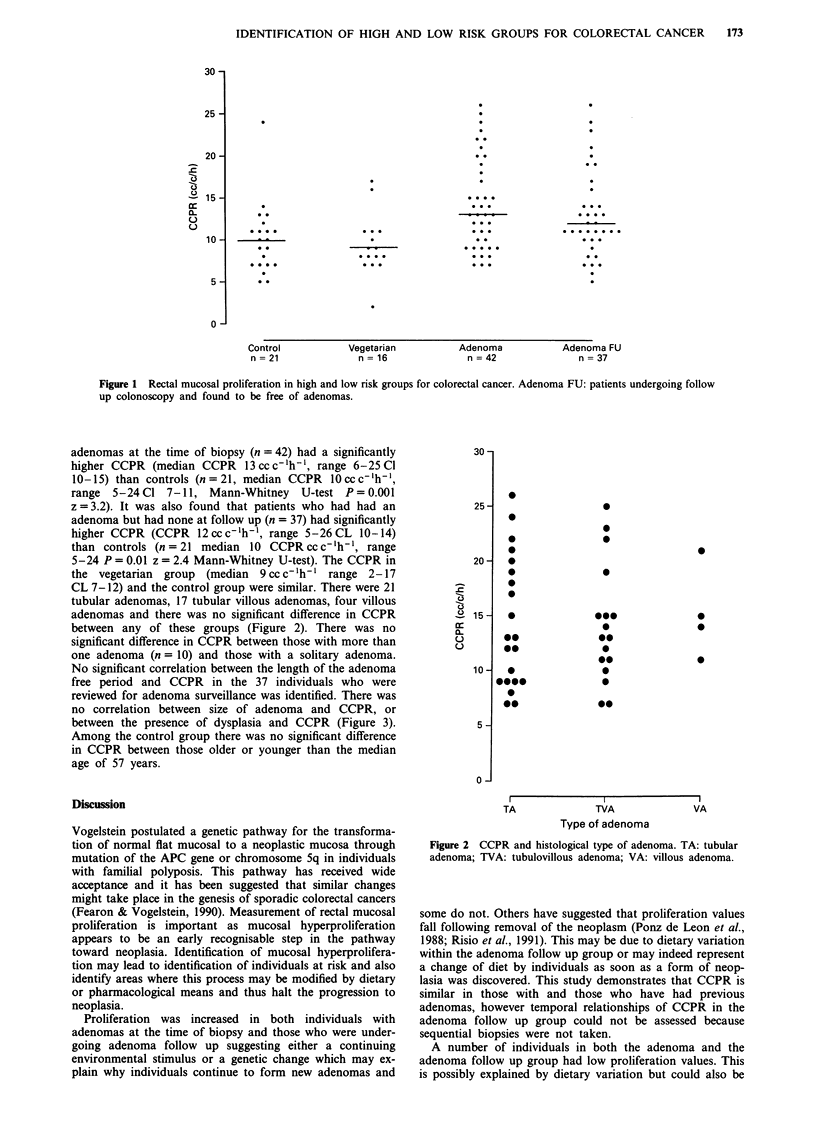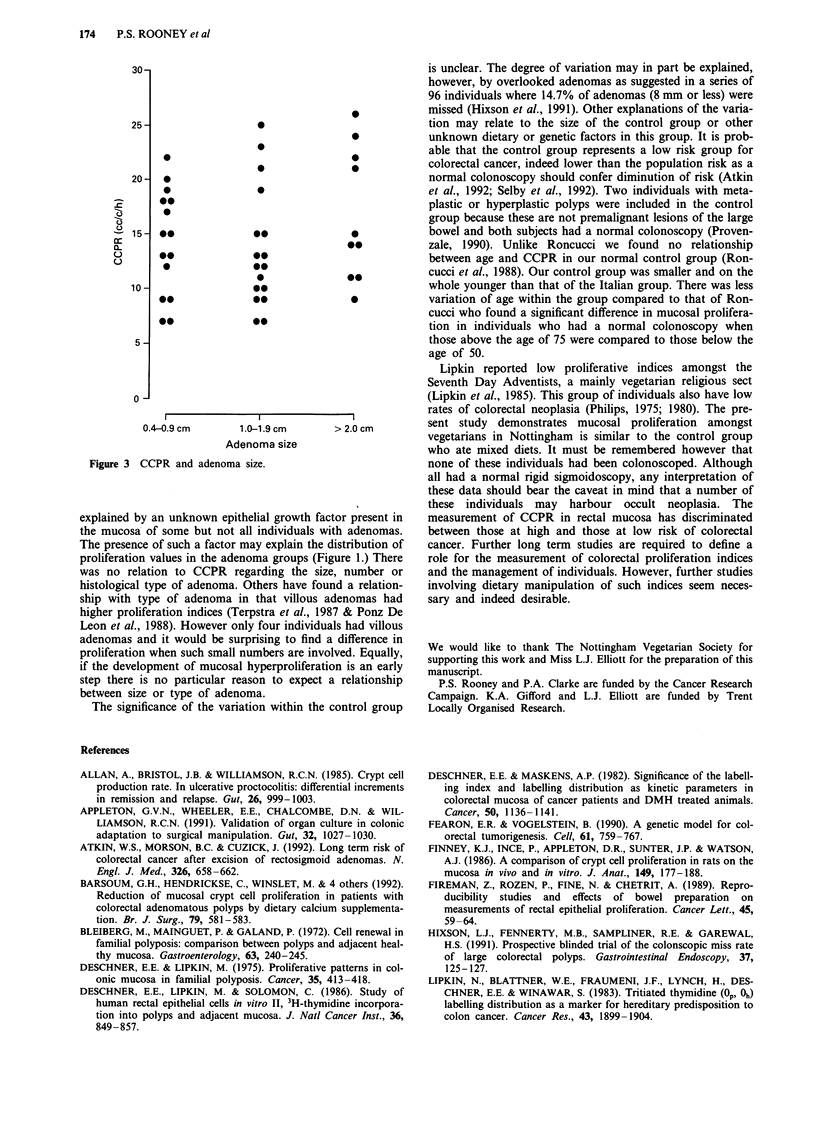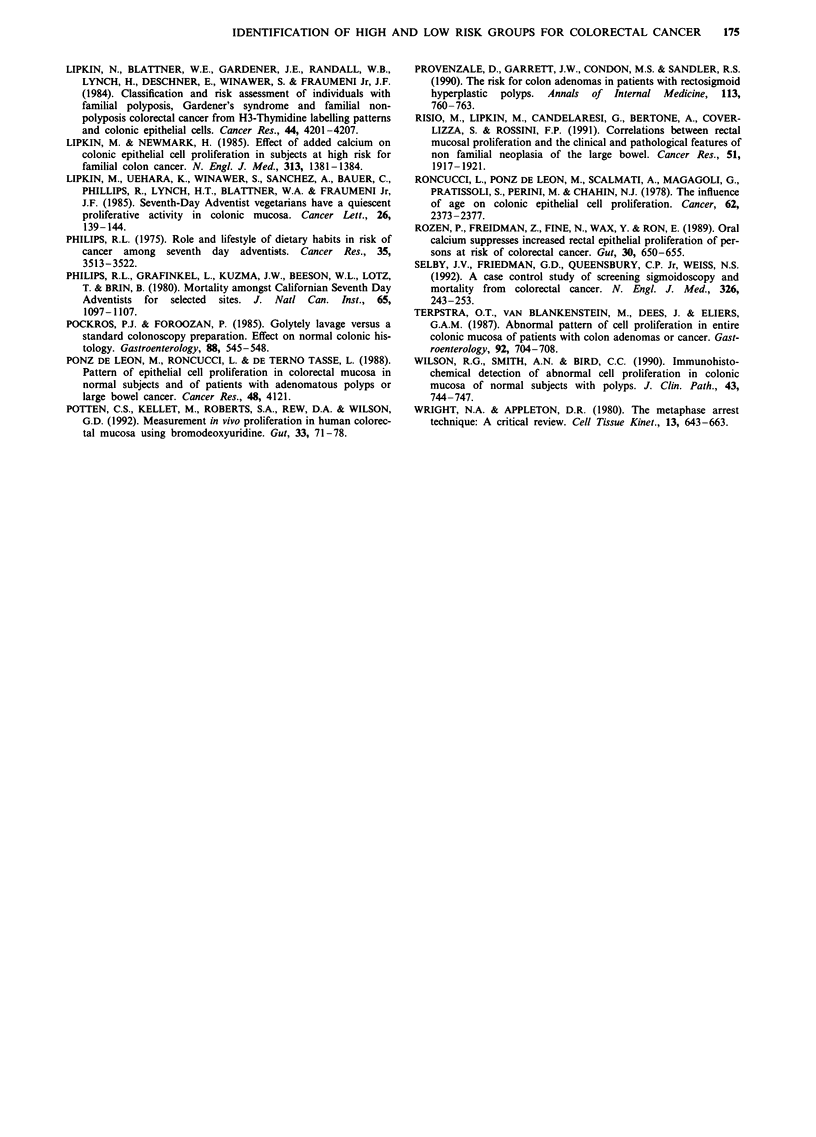# The identification of high and low risk groups for colorectal cancer using rectal mucosal crypt cell production rate (CCPR).

**DOI:** 10.1038/bjc.1993.308

**Published:** 1993-07

**Authors:** P. S. Rooney, P. A. Clarke, K. A. Gifford, J. D. Hardcastle, N. C. Armitage

**Affiliations:** Department of Surgery, University Hospital, Nottingham, UK.

## Abstract

Rectal mucosal proliferation was measured in 116 individuals using the metaphase arrest technique crypt cell production rate (CCPR). CCPR was found to be significantly elevated in individuals with adenomas (n = 42, CCPR = 13 cc c-1h-1, range 7-25 Cl 10-15) compared with normals (n = 21, CCPR = 10 cc c-1h-1 range 5-24 Cl 7-11, Mann-Whitney P = 0.001 z = 3.2). Mucosal proliferation was increased among individuals who were undergoing adenoma follow up but in whom no further adenomas were found (n = 37 CCPR = 12 range 5-26 cc c-1h-1 Cl 10-14) compared to controls (Mann-Whitney P = 0.01 z = 2.4) Proliferation in vegetarians i.e. low risk (n = 16) was similar to controls. Measurement of proliferative indices in rectal mucosa by the stathmokinetic technique CCPR can discriminate between high and low risk groups for colorectal cancer.


Br. J. Cancer (1993), 68, 172–175 © Macmillan Press Ltd., 1993

### The identification of high and low risk groups for colorectal cancer using rectal mucosal crypt cell production rate (CCPR)

P.S. Rooney, P.A. Clarke, K.A. Gifford, J.D. Hardcastle & N.C. Armitage

*Department of Surgery, University Hospital, Nottingham, NG7 2UH, UK.*

**Summary** Rectal mucosal proliferation was measured in 116 individuals using the metaphase arrest technique crypt cell production rate (CCPR). CCPR was found to be significantly elevated in individuals with adenomas ($n = 42$, CCPR = 13 cc $c^{-1}h^{-1}$, range 7–25 Cl 10–15) compared with normals ($n = 21$, CCPR = 10 cc $c^{-1}h^{-1}$ range 5–24 Cl 7–11, Mann-Whitney $P = 0.001$ z = 3.2). Mucosal proliferation was increased among individuals who were undergoing adenoma follow up but in whom no further adenomas were found ($n = 37$ CCPR = 12 range 5–26 cc $c^{-1}h^{-1}$ Cl 10–14) compared to controls (Mann-Whitney $P = 0.01$ z = 2.4) Proliferation in vegetarians i.e. low risk ($n = 16$) was similar to controls.

Measurement of proliferative indices in rectal mucosa by the stathmokinetic technique CCPR can discriminate between high and low risk groups for colorectal cancer.

There is evidence suggesting that there is a link between mucosal proliferation and colorectal neoplasia (Deschner *et al.*, 1966; Bleiberg *et al.*, 1972; Deschner & Lipkin 1975; Deschner & Maskens 1982; Lipkin *et al.*, 1983; Lipkin *et al.*, 1984). The value of measuring mucosal proliferation has assumed further importance since dietary supplementation with calcium was found to reduce the proliferative index in groups of individuals at increased risk of colorectal cancer (Lipkin & Newmark 1985; Rozen *et al.*, 1989). A number of methods are available to measure mucosal proliferation (Lipkin *et al.*, 1983; Wilson *et al.*, 1990; Risio *et al.*, 1991; Potten *et al.*, 1992). *In vitro* labelling with tritiated thymidine is time consuming and not suitable for large numbers of samples. Wright proposed the stathmokinetic technique CCPR, as a more suitable method for measuring mucosal proliferation. The technique identifies the proliferative compartment in the colonic crypt by arresting dividing cells in metaphase using vincristine (Wright & Appleton, 1980). The technique identifies the whole of the crypt so taking into account compartment size and avoiding errors due sectioning found in immunohistochemical techniques (Risio *et al.*, 1991; Wilson *et al.*, 1991). A recent report demonstrated that crypt cell production rate could identify individuals with adenomas and earlier Allan used the technique in measuring proliferative changes in ulcerative colitis (Allan *et al.*, 1985; Barsoum *et al.*, 1992).

The aim of the present study was to use measurements of CCPR to determine individuals who may be at an increased or decreased risk of colorectal neoplasia because of the presence of adenomas.

### Patients and methods

We studied 116 patients, 54 men, 62 women aged between 20–75 years (median 56 years). There were 42 individuals with adenomas at the time of biopsy (24 newly diagnosed and 18 having had previous adenomas), 37 asymptomatic individuals undergoing colonoscopic surveillance for adenomas who were found to be neoplasia free (median adenoma free period 12 months (range 6–72)), 16 Vegetarian asymptomatic volunteers of long standing (> 10 years) (14 lacto vegetarians and two vegans) with no family history or personal history of colorectal cancer who did not undergo colonoscopy and 21 individuals with no family history of colorectal cancer who presented with symptoms of large bowel disease ($n = 11$) or positive faecal occult bloods but had a normal colonoscopy ($n = 8$) or only metaplastic polyps ($n = 2$). This group represented a low risk group. No individual in this study had colorectal cancer prior to or at the time of biopsy. Ethical approval was given by the local ethical committee.

Endoscopic rectal biopsies were taken from each patient 8 cm from the anal margin and at least 5 cm from any macroscopic lesion. The standard bowel preparation used was Klean Prep (polyethylene glycol, Norgine Ltd, UK) because unlike senna preparations it has little effect on histology and does not affect mucosal proliferation (Pockros & Foroozan 1985; Fireman *et al.*, 1989). Vegetarians did not have any bowel preparation before the biopsy. Normal histological mucosa was verified in every case; dysplasia and type of adenoma were assessed without knowledge of proliferation data. Where more than one adenoma was found ($n = 10$) the largest was cited for statistical analysis. Assessment of mucosal proliferation was carried out without knowledge of colonoscopic or histological findings.

Samples were placed in culture medium, RPMI 1640, Gibco Ltd and transferred to the laboratory. Tissue was stored in supplemented RPMI 1640 and gentamycin 0.001% (Nicholas Pharmaceuticals) for 16 h to avoid extraction artefact (Finney *et al.*, 1986; Appleton *et al.*, 1991). At the time of assay the culture medium was replaced with fresh medium containing 1 ml of 5 $\mu g\,ml^{-1}$ of vincristine. The culture medium containing samples was incubated in an atmosphere of 5% carbon dioxide and 95% oxygen. The samples were removed from tissue culture at 25, 50 and 75 min time points, fixed in Carnoy's solution and stained in Schiffs reagent, a similar method to that of Barsoum (Barsoum *et al.*, 1992). The number of metaphase arrests was counted in 20–30 crypts per sample. CCPR was calculated from the three time points by least squares regression analysis and expressed in crypt cells per crypt per hour (cc $c^{-1}h^{-1}$).

### Statistical analysis

Proliferation data were compared using non-parametric analysis, the Mann-Whitney U-test. Comparisons of age were assessed by the unpaired Student's *t*-test. Confidence limits were calculated at the 95% level.

### Results

Biopsies were collected from 116 patients. Differences in median CCPR are shown in Figure 1. Patients with

Correspondence: P.S. Rooney, Department of Surgery, University Hospital, Nottingham, NG7 2UH, UK.
Received 24 November 1992; and in revised form 4 February 1993.

**Figure 1** Rectal mucosal proliferation in high and low risk groups for colorectal cancer. Adenoma FU: patients undergoing follow up colonoscopy and found to be free of adenomas.

adenomas at the time of biopsy ($n = 42$) had a significantly higher CCPR (median CCPR 13 cc $c^{-1}h^{-1}$, range 6–25 Cl 10–15) than controls ($n = 21$, median CCPR 10 cc $c^{-1}h^{-1}$, range 5–24 Cl 7–11, Mann-Whitney U-test $P = 0.001$ $z = 3.2$). It was also found that patients who had had an adenoma but had none at follow up ($n = 37$) had significantly higher CCPR (CCPR 12 cc $c^{-1}h^{-1}$, range 5–26 CL 10–14) than controls ($n = 21$ median 10 CCPR cc $c^{-1}h^{-1}$, range 5–24 $P = 0.01$ $z = 2.4$ Mann-Whitney U-test). The CCPR in the vegetarian group (median 9 cc $c^{-1}h^{-1}$ range 2–17 CL 7–12) and the control group were similar. There were 21 tubular adenomas, 17 tubular villous adenomas, four villous adenomas and there was no significant difference in CCPR between any of these groups (Figure 2). There was no significant difference in CCPR between those with more than one adenoma ($n = 10$) and those with a solitary adenoma. No significant correlation between the length of the adenoma free period and CCPR in the 37 individuals who were reviewed for adenoma surveillance was identified. There was no correlation between size of adenoma and CCPR, or between the presence of dysplasia and CCPR (Figure 3). Among the control group there was no significant difference in CCPR between those older or younger than the median age of 57 years.

**Figure 2** CCPR and histological type of adenoma. TA: tubular adenoma; TVA: tubulovillous adenoma; VA: villous adenoma.

### Discussion

Vogelstein postulated a genetic pathway for the transformation of normal flat mucosal to a neoplastic mucosa through mutation of the APC gene or chromosome 5q in individuals with familial polyposis. This pathway has received wide acceptance and it has been suggested that similar changes might take place in the genesis of sporadic colorectal cancers (Fearon & Vogelstein, 1990). Measurement of rectal mucosal proliferation is important as mucosal hyperproliferation appears to be an early recognisable step in the pathway toward neoplasia. Identification of mucosal hyperproliferation may lead to identification of individuals at risk and also identify areas where this process may be modified by dietary or pharmacological means and thus halt the progression to neoplasia.

Proliferation was increased in both individuals with adenomas at the time of biopsy and those who were undergoing adenoma follow up suggesting either a continuing environmental stimulus or a genetic change which may explain why individuals continue to form new adenomas and some do not. Others have suggested that proliferation values fall following removal of the neoplasm (Ponz de Leon *et al.*, 1988; Risio *et al.*, 1991). This may be due to dietary variation within the adenoma follow up group or may indeed represent a change of diet by individuals as soon as a form of neoplasia was discovered. This study demonstrates that CCPR is similar in those with and those who have had previous adenomas, however temporal relationships of CCPR in the adenoma follow up group could not be assessed because sequential biopsies were not taken.

A number of individuals in both the adenoma and the adenoma follow up group had low proliferation values. This is possibly explained by dietary variation but could also be

**Figure 3** CCPR and adenoma size.

explained by an unknown epithelial growth factor present in the mucosa of some but not all individuals with adenomas. The presence of such a factor may explain the distribution of proliferation values in the adenoma groups (Figure 1.) There was no relation to CCPR regarding the size, number or histological type of adenoma. Others have found a relationship with type of adenoma in that villous adenomas had higher proliferation indices (Terpstra *et al.*, 1987 & Ponz De Leon *et al.*, 1988). However only four individuals had villous adenomas and it would be surprising to find a difference in proliferation when such small numbers are involved. Equally, if the development of mucosal hyperproliferation is an early step there is no particular reason to expect a relationship between size or type of adenoma.

The significance of the variation within the control group is unclear. The degree of variation may in part be explained, however, by overlooked adenomas as suggested in a series of 96 individuals where 14.7% of adenomas (8 mm or less) were missed (Hixson *et al.*, 1991). Other explanations of the variation may relate to the size of the control group or other unknown dietary or genetic factors in this group. It is probable that the control group represents a low risk group for colorectal cancer, indeed lower than the population risk as a normal colonoscopy should confer diminution of risk (Atkin *et al.*, 1992; Selby *et al.*, 1992). Two individuals with metaplastic or hyperplastic polyps were included in the control group because these are not premalignant lesions of the large bowel and both subjects had a normal colonoscopy (Provenzale, 1990). Unlike Roncucci we found no relationship between age and CCPR in our normal control group (Roncucci *et al.*, 1988). Our control group was smaller and on the whole younger than that of the Italian group. There was less variation of age within the group compared to that of Roncucci who found a significant difference in mucosal proliferation in individuals who had a normal colonoscopy when those above the age of 75 were compared to those below the age of 50.

Lipkin reported low proliferative indices amongst the Seventh Day Adventists, a mainly vegetarian religious sect (Lipkin *et al.*, 1985). This group of individuals also have low rates of colorectal neoplasia (Philips, 1975; 1980). The present study demonstrates mucosal proliferation amongst vegetarians in Nottingham is similar to the control group who ate mixed diets. It must be remembered however that none of these individuals had been colonoscoped. Although all had a normal rigid sigmoidoscopy, any interpretation of these data should bear the caveat in mind that a number of these individuals may harbour occult neoplasia. The measurement of CCPR in rectal mucosa has discriminated between those at high and those at low risk of colorectal cancer. Further long term studies are required to define a role for the measurement of colorectal proliferation indices and the management of individuals. However, further studies involving dietary manipulation of such indices seem necessary and indeed desirable.

We would like to thank The Nottingham Vegetarian Society for supporting this work and Miss L.J. Elliott for the preparation of this manuscript.

P.S. Rooney and P.A. Clarke are funded by the Cancer Research Campaign. K.A. Gifford and L.J. Elliott are funded by Trent Locally Organised Research.

### References

ALLAN, A., BRISTOL, J.B. & WILLIAMSON, R.C.N. (1985). Crypt cell production rate. In ulcerative proctocolitis: differential increments in remission and relapse. *Gut*, **26**, 999–1003.

APPLETON, G.V.N., WHEELER, E.E., CHALCOMBE, D.N. & WILLIAMSON, R.C.N. (1991). Validation of organ culture in colonic adaptation to surgical manipulation. *Gut*, **32**, 1027–1030.

ATKIN, W.S., MORSON, B.C. & CUZICK, J. (1992). Long term risk of colorectal cancer after excision of rectosigmoid adenomas. *N. Engl. J. Med.*, **326**, 658–662.

BARSOUM, G.H., HENDRICKSE, C., WINSLET, M. & 4 others (1992). Reduction of mucosal crypt cell proliferation in patients with colorectal adenomatous polyps by dietary calcium supplementation. *Br. J. Surg.*, **79**, 581–583.

BLEIBERG, M., MAINGUET, P. & GALAND, P. (1972). Cell renewal in familial polyposis: comparison between polyps and adjacent healthy mucosa. *Gastroenterology*, **63**, 240–245.

DESCHNER, E.E. & LIPKIN, M. (1975). Proliferative patterns in colonic mucosa in familial polyposis. *Cancer*, **35**, 413–418.

DESCHNER, E.E., LIPKIN, M. & SOLOMON, C. (1986). Study of human rectal epithelial cells *in vitro* II, $^3$H-thymidine incorporation into polyps and adjacent mucosa. *J. Natl Cancer Inst.*, **36**, 849–857.

DESCHNER, E.E. & MASKENS, A.P. (1982). Significance of the labelling index and labelling distribution as kinetic parameters in colorectal mucosa of cancer patients and DMH treated animals. *Cancer*, **50**, 1136–1141.

FEARON, E.R. & VOGELSTEIN, B. (1990). A genetic model for colorectal tumorigenesis. *Cell*, **61**, 759–767.

FINNEY, K.J., INCE, P., APPLETON, D.R., SUNTER, J.P. & WATSON, A.J. (1986). A comparison of crypt cell proliferation in rats on the mucosa *in vivo* and *in vitro*. *J. Anat.*, **149**, 177–188.

FIREMAN, Z., ROZEN, P., FINE, N. & CHETRIT, A. (1989). Reproducibility studies and effects of bowel preparation on measurements of rectal epithelial proliferation. *Cancer Lett.*, **45**, 59–64.

HIXSON, L.J., FENNERTY, M.B., SAMPLINER, R.E. & GAREWAL, H.S. (1991). Prospective blinded trial of the colonscopic miss rate of large colorectal polyps. *Gastrointestinal Endoscopy*, **37**, 125–127.

LIPKIN, N., BLATTNER, W.E., FRAUMENI, J.F., LYNCH, H., DESCHNER, E.E. & WINAWAR, S. (1983). Tritiated thymidine ($0_p$, $0_h$) labelling distribution as a marker for hereditary predisposition to colon cancer. *Cancer Res.*, **43**, 1899–1904.

LIPKIN, N., BLATTNER, W.E., GARDENER, J.E., RANDALL, W.B., LYNCH, H., DESCHNER, E., WINAWER, S. & FRAUMENI Jr, J.F. (1984). Classification and risk assessment of individuals with familial polyposis, Gardener's syndrome and familial non-polyposis colorectal cancer from H3-Thymidine labelling patterns and colonic epithelial cells. *Cancer Res.*, **44**, 4201–4207.

LIPKIN, M. & NEWMARK, H. (1985). Effect of added calcium on colonic epithelial cell proliferation in subjects at high risk for familial colon cancer. *N. Engl. J. Med.*, **313**, 1381–1384.

LIPKIN, M., UEHARA, K., WINAWER, S., SANCHEZ, A., BAUER, C., PHILLIPS, R., LYNCH, H.T., BLATTNER, W.A. & FRAUMENI Jr, J.F. (1985). Seventh-Day Adventist vegetarians have a quiescent proliferative activity in colonic mucosa. *Cancer Lett.*, **26**, 139–144.

PHILIPS, R.L. (1975). Role and lifestyle of dietary habits in risk of cancer among seventh day adventists. *Cancer Res.*, **35**, 3513–3522.

PHILIPS, R.L., GRAFINKEL, L., KUZMA, J.W., BEESON, W.L., LOTZ, T. & BRIN, B. (1980). Mortality amongst Californian Seventh Day Adventists for selected sites. *J. Natl Can. Inst.*, **65**, 1097–1107.

POCKROS, P.J. & FOROOZAN, P. (1985). Golytely lavage versus a standard colonoscopy preparation. Effect on normal colonic histology. *Gastroenterology*, **88**, 545–548.

PONZ DE LEON, M., RONCUCCI, L. & DE TERNO TASSE, L. (1988). Pattern of epithelial cell proliferation in colorectal mucosa in normal subjects and of patients with adenomatous polyps or large bowel cancer. *Cancer Res.*, **48**, 4121.

POTTEN, C.S., KELLET, M., ROBERTS, S.A., REW, D.A. & WILSON, G.D. (1992). Measurement *in vivo* proliferation in human colorectal mucosa using bromodeoxyuridine. *Gut*, **33**, 71–78.

PROVENZALE, D., GARRETT, J.W., CONDON, M.S. & SANDLER, R.S. (1990). The risk for colon adenomas in patients with rectosigmoid hyperplastic polyps. *Annals of Internal Medicine*, **113**, 760–763.

RISIO, M., LIPKIN, M., CANDELARESI, G., BERTONE, A., COVERLIZZA, S. & ROSSINI, F.P. (1991). Correlations between rectal mucosal proliferation and the clinical and pathological features of non familial neoplasia of the large bowel. *Cancer Res.*, **51**, 1917–1921.

RONCUCCI, L., PONZ DE LEON, M., SCALMATI, A., MAGAGOLI, G., PRATISSOLI, S., PERINI, M. & CHAHIN, N.J. (1978). The influence of age on colonic epithelial cell proliferation. *Cancer*, **62**, 2373–2377.

ROZEN, P., FREIDMAN, Z., FINE, N., WAX, Y. & RON, E. (1989). Oral calcium suppresses increased rectal epithelial proliferation of persons at risk of colorectal cancer. *Gut*, **30**, 650–655.

SELBY, J.V., FRIEDMAN, G.D., QUEENSBURY, C.P. Jr, WEISS, N.S. (1992). A case control study of screening sigmoidoscopy and mortality from colorectal cancer. *N. Engl. J. Med.*, **326**, 243–253.

TERPSTRA, O.T., VAN BLANKENSTEIN, M., DEES, J. & ELIERS, G.A.M. (1987). Abnormal pattern of cell proliferation in entire colonic mucosa of patients with colon adenomas or cancer. *Gastroenterology*, **92**, 704–708.

WILSON, R.G., SMITH, A.N. & BIRD, C.C. (1990). Immunohistochemical detection of abnormal cell proliferation in colonic mucosa of normal subjects with polyps. *J. Clin. Path.*, **43**, 744–747.

WRIGHT, N.A. & APPLETON, D.R. (1980). The metaphase arrest technique: A critical review. *Cell Tissue Kinet.*, **13**, 643–663.